# Understanding the experiences of receiving and providing maternity care for autistic adults: A Multi-perspectival Interpretative Phenomenological Analysis study

**DOI:** 10.1177/13623613241274518

**Published:** 2024-09-06

**Authors:** Laura Moore, Sarah Foley, Fionnuala Larkin

**Affiliations:** University College Cork, Ireland

**Keywords:** adults, autism spectrum disorders, health services, qualitative research

## Abstract

Available research suggests autistic mothers experience greater dissatisfaction with maternity care compared to non-autistic mothers. Limited research exists exploring autistic mothers’ experiences of maternity care, and no research to date has considered maternity care professionals’ experiences of delivering care to autistic mothers. This qualitative study aims to explore the experiences of receiving and providing maternity care for autistic mothers. Data were collected using semi-structured interviews with four autistic mothers and four midwives. Data were analysed using Multi-perspectival Interpretative Phenomenological Analysis. Three overarching themes emerged: ‘challenges faced within maternity care, ‘the importance of the caring relationship’, and ‘the challenge of navigating competing demands and priorities’. Findings highlight the unique challenges experienced by autistic mothers during labour and birth, the need for increased autism awareness in midwifery, and further suggest universal changes to the delivery of maternity care which would benefit autistic women.

**Lay abstract:**

Autistic mothers may experience unique challenges when accessing maternity care. A better understanding of the experience of autistic mothers and maternity care professionals would help to create opportunities to support better maternity care. In this study, we interviewed autistic mothers and professional midwives, living and working across the United Kingdom and Ireland. In the interviews, the autistic mothers recalled challenges they faced in the hospital settings, difficulties in communicating their needs, and distress when being physically examined. The midwives we interviewed brought their personal experiences of autism (some were autistic themselves, while others had autistic family members) and made efforts to accommodate autistic mothers where possible. This included paying attention to potential sensory issues, trying to establish a relationship with the mothers and communicating what was going on without medical jargon. However, the midwives were limited in their ability to fully attend to the needs of autistic mothers due to time and resource restraints. Both the midwives and autistic mothers felt that midwife-led births were more attentive to the needs of mothers. Based on our findings, we recommend further training and awareness on autism in midwifery and suggest that changes relating to sensory and communication challenges would benefit both autistic and non-autistic. Our study provides important insight into this experience of maternity care from two perspectives and emphasises the need for greater inclusivity in maternity care services.

The perinatal period (pregnancy and 1 month post-birth) has been identified as a critical stage for a range of maternal and infant health and well-being outcomes (Misra et al., 2003). Despite calls for the prioritisation of research on women and girls by the autism community (Pellicano et al., 2014), and the increase in women with a diagnosis (Loomes et al., 2017), there remains limited research exploring the experience of the perinatal period for autistic women (McDonnell & DeLucia, 2021). One epidemiological study found evidence that autistic mothers are at greater risk of preterm births and caesarean delivery (Sundelin et al., 2018). Pohl et al. (2020) conducted a survey study comparing the perinatal experiences of autistic mothers and non-autistic mothers, finding that autistic mothers reported greater incidence of antenatal and postnatal depression, increased anxiety and isolation, and difficulty seeking support. This has been further supported by a UK-based study (Hampton et al., 2022), indicating that autistic people may have unique experiences and challenges in the perinatal period which warrant further research.

## Challenges in the perinatal period for autistic mothers

Despite the limited nature of the available research, two clear themes have emerged regarding the specific challenges of the perinatal period for autistic people. Previous studies have found that the sensory aspects of pregnancy and childbirth can be challenging (Donovan, 2020; Gardner et al., 2016; Hampton et al., 2022; Rogers et al., 2017). This literature noted that autistic people felt as though their pre-existing sensory processing issues were heightened during pregnancy (Hampton et al., 2022, 2024). For example, attending busy maternity clinics with bright lights and noise can be a source of distress, and having regular physical examinations can also be particularly stressful (Samuel et al., 2022). Autistic people also report more difficulties than non-autistic people with the bodily changes associated with pregnancy (e.g. nausea, pelvic girdle pain), and experience higher levels of meltdowns and shutdowns (Hampton et al., 2024). Furthermore, a survey study by Pohl et al. (2020) reported higher rates of pre- or post-partum depression in autistic mothers compared to non-autistic mothers, and greater self-reported difficulties in parenting tasks such as multi-tasking and domestic work.

Difficulties with communication and interactions with professionals have also been consistently reported. Autistic mothers reported greater dissatisfaction with their communication with health care professionals during pregnancy (Pohl et al., 2020). In a wider study of health communication with autistic women, Lum et al. (2014) found that autistic adults reported dissatisfaction with information and support services provided in pregnancy, and greater difficulty with communicating about pain, concerns or needs, during childbirth (Lum et al., 2014). This implies that there are specific communication difficulties within the relationship between autistic mothers and maternity care professionals (MCPs), which warrant further exploration. There is a developing qualitative literature on the experience of the perinatal period for autistic women that details first-person accounts and reflections on the perinatal experience (Donovan et al., 2023; Hampton et al., 2024; Rogers et al., 2017; Talcer et al., 2023). Talcer et al. (2023) echo previous research on the impact of sensory overload and highlights the importance of offering adaptations to procedures during labour and birth. A thematic analysis of 21 autistic mothers’ experiences of pregnancy, postnatal support, and parenting (Hampton et al., 2022) substantiates findings from quantitative research and suggests the importance of communication and relationships with health care professionals in a maternity care context. Further qualitative studies include an examination of pre-existing blogs (Litchman et al., 2019), secondary analysis of online Asperger’s support groups (Gardner et al., 2016), and an interpretative descriptive account of autistic mothers’ childbirth experiences in an acute setting (Donovan, 2020). Findings from qualitative studies again highlight the importance autistic mothers place on being able to build a relationship and communicate well with MCPs (Donovan, 2020; Gardner et al., 2016; Hampton et al., 2022), without which mothers can feel distrustful of nurses and judged as mothers (Donovan et al., 2023). Given that prior research within a neurotypical population reports that women who felt ‘very well’ cared for in the perinatal period are likely to experience improved postnatal functioning (Michels et al., 2013), it is imperative that this experience of maternity care is understood for autistic women specifically.

## Maternity care professionals and autism

Research on MCPs’ knowledge and options in providing maternity care for autistic people is lacking (Hampton et al., 2022). To date, research with other groups of health care professionals, including GPs, nurses, and allied health professionals (Morris et al., 2019; Urbanowicz et al., 2020; Zerbo et al., 2015) has reported only low to moderate knowledge of autism (Corden et al., 2021). This is further supported in a UK survey study with GPs, who reported they had limited confidence in their ability to identify and appropriately manage autistic patients, despite having good knowledge of autism (Unigwe et al., 2017). This lack of confidence and self-efficacy likely contributes to the interaction difficulties which have been noted as a barrier for autistic people accessing health care services (Dern & Sappok, 2016). A survey of 355 autistic mothers found that 80% worried about disclosing an autism diagnosis to health care professionals, and 40% rarely or never used services for autistic people (Department of Health, 2010; National Disability Authority, 2014). Pregnant autistic women in a qualitative study reported that they felt uncomfortable discussing their diagnosis with MCPs (Hampton et al., 2024). There is no specific consideration of, or guidelines around, maternity care for autistic people, highlighting the need for further research in this domain.

## The present study

The current study aims to deepen the understanding of the experience of maternity care for autistic mothers, with a particular focus on making sense of their interactions with MCPs. As, to the best of our knowledge, no available research exists exploring MCPs’ experiences of delivering care to autistic women, the study also aims to understand how MCPs make sense of their experiences delivering maternity care to this group of patients. This multi-perspectival approach thus explores the meanings both groups attach to the experience of the relationship, challenges experienced within these interactions, and considerations of good practice in maternity care for autistic mothers.

## Methods

### Study design

A Multi-perspectival Interpretative Phenomenological Analysis (IPA) design was adopted in the current study to explore the experience of maternity care for autistic mothers and midwives. The double hermeneutic and commitment to reflexivity within IPA have marked it as a particularly appropriate qualitative methodology within participatory autism research (MacLeod, 2019). A multi-perspectival design was selected due to the relational and systemic nature of the phenomenon of maternity care. Multi-perspectival IPA maintains an idiographic approach to data collection and analysis but extends this by synthesising analyses within and between samples (Larkin et al., 2019). Multi-perspectival designs offer particular advantage in exploring relationships between patients and health care providers (Borg Xuereb et al., 2015; Larkin et al., 2019).

### Participants

Purposive sampling was used in order to recruit two relatively homogeneous groups (Smith et al., 2009) of four autistic mothers (Table 1) and four MCPs (Table 2). Autistic mothers met the inclusion criteria if they (a) had an autism diagnosis, (b) had given birth, (c) were over 18 and (d) were not experiencing acute mental health symptoms at the time of the interview. No potential participants were excluded. We included only women with a formal diagnosis to account for the need for homogeneity for IPA: we anticipated that women who had all been through the diagnostic process might be more homogeneous.

**Table 1.** Autistic mothers’ demographic information.

Pseudonym	Location	Age	Age of children	Time of autism diagnosis
Ellen	The United Kingdom	45	20	After Perinatal Period
Rebecca	The United Kingdom	38	8 & 4	During Perinatal period
Jennifer	The United Kingdom	37	4	Pre-pregnancy
Alice	Ireland	38	7	After Perinatal Period

**Table 2.** Midwives’ demographic information.

Pseudonym	Location	Age	Mother	Autism status
Katie	The United Kingdom	25	No	Autistic
Ann	The United Kingdom	48	Yes	Not autistic
Maggie	The United Kingdom	34	Yes	Autistic
Sue	The United Kingdom	29	No	Not autistic

Professionals met the inclusion criteria if they (a) had experience of working within maternity care and (b) had provided maternity care to people on the autism spectrum. All participants within the professional sample worked as midwives. Importantly, two of the midwives had a diagnosis of autism. The other two midwives had strong personal connections with autism, with one having a daughter on the autism spectrum and the other having an autistic brother. This sample of midwives had a clear personal interest in autism and the delivery of maternity care to autistic people and may not be representative of the views and experiences of midwifery as a whole. Socioeconomic status and educational status were not recorded. All participants were white European women.

### Procedure

Institutional ethical approval was obtained for the study. Participants were recruited via social media. Potential participants contacted the lead researcher and were sent an information sheet and returned a consent form. A semi-structured interview schedule for autistic mothers was designed in collaboration with the Public Patient Involvement (PPI) panel (see Community Involvement). A separate semi-structured interview schedule was designed in order to access the experiences of MCPs. All participants in the current study opted for online interviews and varied in their preference for having their cameras on or off. Interviews lasted between 35 and 75 min.

### Data analysis

The multi-perspectival IPA analysis was informed by the inductive and iterative approach outlined by Smith and Nizza (2022) and key multi-perspectival papers by Rostill-Brookes et al. (2011) and Larkin et al. (2019). Each individual transcript was read and re-read, and exploratory descriptive, linguistic, and conceptual notes were made. Then, experiential statements were formulated to capture the idiographic meaning of participants’ experiences within each portion of individual interviews. Next, experiential statements were clustered for each individual interview in order to find patterns of connection within experiential statements. A table of personal experiential statements was then compiled for each individual participants’ interview. Next, cross-case analysis within the group of autistic mothers and within the group of midwives was conducted, resulting in experiential themes for each group. The final step in the multi-perspectival analysis was looking for relationships, convergence and divergence between the two groups. This step included relabelling some of the themes in order to consider the relationship between groups and resulted in the production of a final table of themes.

### Community involvement

None of the authors of the current study are autistic, and therefore took measures to ensure appropriate study design and analysis was conducted that met the needs of autistic participants. The study design was informed by a project advisory panel of autistic people, utilising a consultation approach to PPI research (INVOLVE, 2012). The panel consisted of five autistic mothers, all based in the United Kingdom, aged between 25 and 45. An online meeting was held with the group to discuss the study rationale and methods, and drafts of documents were sent to them for review and comments. The PPI panel gave feedback and advice on the design of interview schedules, information sheets and on data collection procedures. The panel were paid for their time.

## Results

**Figure 1.**
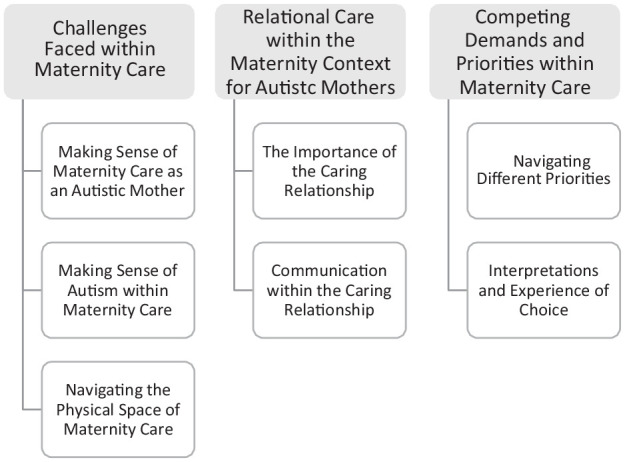
Themes and subthemes (Smith et al., 2009).

Multi-perspectival analysis resulted in three major themes and respective subthemes (Figure 1).

### Challenges faced within maternity care

Both mothers and midwives experienced challenges when making sense of how autistic people ‘fit’ within a maternity care system. Both groups also described the challenge of the sensory aspects of maternity care for autistic mothers.

#### Making sense of maternity care as an autistic mother

For mothers on the autism spectrum, the experience of maternity care was often confusing and overwhelming. Labour was described as particularly overwhelming:

I felt very frightened almost and just confused . . . I was just bewildered by the whole process. (Ellen)

Autistic mothers struggled to make sense of their experience when their own expectations were not met. Jennifer describes how she struggled when her expectations did not match her experience during the initial period when she brought her son home:

But in that moment, I think I was the most rigid black and white I’ve ever been. I’ve been very much, child cries he’s fed, he doesn’t have a wet diaper, he must either be in pain or hate me.

As autistic mothers were trying to make sense of the experience of maternity care, they were also trying to make sense of and communicate their own support needs. Alice described that ‘knowing myself better’ would have improved her experience of maternity care. This sense was shared by Rebecca, who felt better able to understand and, thus, express her needs in her second pregnancy:

‘Yeah, second time around there’s very small specifics, whereas first time around was a mixture of naivete and not really knowing.

This subtheme highlights the challenging role that expectations play within maternity care for autistic women. Autistic women’s maternity experiences are complicated by the need to make sense of how their autism-specific needs are impacting their perception of what is an already challenging experience.

#### Making sense of autism within maternity care as a midwife

The midwives sensed that autism is an overlooked experience within maternity care, as explained by Maggie: ‘There’s nothing at all. There are no policies in the hospital. There’s just nothing in place whatsoever . . .’. This lack of consideration to autism was understood to have negative implications for patients:

I don’t want to say the word discriminated, but sometimes I think they probably are because they’re not being looked after in the best way possible, and that is impacting their outcomes. (Sue, midwife)

All midwives had a strong personal understanding of autism, which they described as helpful in delivering care to autistic women:

I guess, it’s for me . . . it’s easier to care because I know that they can really identify with a lot of what I’m going through. (Katie, midwife)

Their personal understanding of autism meant the midwives in the current study positioned themselves outside of the dominant understanding of autism within maternity care. This led to challenges in navigating the prevailing attitudes and misperceptions that exist. Here, Ann makes sense of how midwives perceive ‘out of the ordinary’ care choices made by autistic mothers, in this case, questioning the benefits of antibiotics prescribed to their child:

Because . . . they’ve made an informed decision. Whereas that might, maybe sometimes get seen as not advocating for themselves or the baby . . .

Here, the midwife outlines how questions or requests for non-intervention during labour and postnatal care may be misinterpreted as neglectful rather than a request for clarity. This subtheme highlights the challenge of how autistic mothers’ needs fit within a system in which autism is overlooked and misperceptions exist. It also highlights the challenges and opportunities that exist for midwives who have a special interest in autism within maternity care.

#### Navigating the physical space of maternity care

Autistic mothers highlighted the challenges they face in navigating the physical and sensory space of maternity care environments. The sensory sensitivities of autism exacerbated these autistic mothers’ sense of overwhelm and powerlessness within the maternity care context. Ellen describes her experience of being on the labour ward:

. . . the bright lights and the noises and it was, I mean . . . I don’t take drugs, but I imagine when people talk about like having a really out of control trip experience (Ellen, mother)

This highlights the experience of feeling out of control as a result of the intense sensory experiences in the hospital. Mothers found shared wards especially challenging due to sensory overwhelm and the social pressure of shared spaces at a vulnerable time.

Similarly, midwives described their understanding that the unsuitability of the environment can impact on how autistic women present, which feasibly then impacts how they are perceived and treated by MCPs:

If you’re on a busy ward and there’s a lot of noise and things like that it can be really overwhelming for certain people and then they can’t quite control their emotions as well, where they often are going into some sort of meltdown. (Maggie, midwife)

Importantly, midwives highlighted that the physical space of maternity care presents challenges for all mothers, not just those on the autism spectrum:

Before I knew about autism, I would go into a room and I would say things like ‘oh babies don’t like bright lights’ . . . that’s the way I understand birth works best. (Ann, midwife)

Therefore, although the maternity care environment may be experienced as inappropriate for all mothers, the negative impact may be heightened for those on the autism spectrum due to sensory sensitivities. This awareness of sensory overwhelm as a result of environmental factors was shared by mothers and midwives.

### Relational care within the maternity context for autistic mothers

This theme focuses on the central role of the caring relationship within maternity care. Both the autistic mothers and the midwives in this study valued this relationship highly. However, both groups experienced challenges in establishing a caring relationship.

#### The importance of the caring relationship

The autistic mothers in this study experienced a strong desire to build relationships with MCPs and found it challenging to deal with different MCPs at each antenatal appointment:

I didn’t feel like I could have that close enough relationship for someone to see that actually I might have needed a little bit extra . . . (Rebecca, mother)

Both groups believed that continuity of care allowed the professional to identify a mother’s needs.

When mothers perceived a person-centred relationship, this had a positive impact on their overall experience. Below, Ellen describes her high regard for the midwife who conducted home visits after the birth of her child:

And my health visitor was a gift from God . . . that was the first time I’d had anybody who I felt was interested in me as a person, not just you know, a medical specimen. (Ellen, mother)

For the midwives, this ability to build relationships was experienced as a valued and rewarding aspect of their professional identity. Katie noted ‘I feel very protective over the women that are autistic that I’m looking after . . . I will go in and have a lovely chat and bond with her’.

In describing how she experienced supportive interactions with professionals as distinct from challenging interactions, Jennifer described,

The ones that were really good listened to me specifically, understood my issues that were autism related and those that were trauma related and were able to differentiate between the two.

Interpretation of personality also played a role in how midwives made sense of the experience of establishing relationships with autistic mothers. Here, Maggie is describing her perception of colleagues’ difficulties in establishing such relationships: ‘Honestly . . . the worst thing is they don’t really give the women the time, and because they’re more difficult, they seem to give them even less time because that’s how they perceive them as being’.

Therefore, although there is significant value and meaning attached to the caring relationship, there are challenges in establishing this relationship within a maternity care context for autistic mothers.

#### Communication within the caring relationship

Mothers described experiencing challenges in making their needs known to MCPs during the labour process, as described by Ellen:

I felt very internalized in the whole thing . . . and I kind of did shut down in terms of communicating to other people.

This challenge was also evident for the midwives in the sample. Maggie stated, ‘You can find that they don’t really know what to say [during labour]. . . or they’re not just taking anything in, ‘cause they are just shutting down completely’. Some of this challenge was attributed to the prevalence of masking among autistic women:

I was used to minimizing things when I described them, but I needed to accurately describe it, ‘cause I really needed advice but conveying how things actually were . . . I found really difficult’. (Alice, mother, on antenatal appointments)

According to the midwives’ experience, autistic mothers often asked more questions and needed more detail in order to understand ‘routine’ procedures, which negatively impacted how they were perceived by MCPs. Katie describes observing an interaction between an obstetrician and an autistic patient, ‘he talked to her as if she was stupid . . .’ (Katie, midwife).

Communication from professionals was interpreted as overly casual, considering the emotional impact of the information being shared. In making sense of her experience of an internal examination, Rebecca said, ‘Yeah, especially as I was not told that was going to happen at all. She just said she was *just* having a look and she did it without me even . . .’.

Miscommunication was a common feature of the care relationship between autistic mothers and midwives. This quote from Ellen suggests a literal interpretation of communication from a male obstetrician:

He said, ‘I know this is a bit uncomfortable’ and I went, ‘No, you don’t’ . . . you’ve got absolutely no idea what it’s like. . . (Ellen, mother)

The midwives within this study felt an important part of their role was bridging the gap between other MCPs and autistic mothers, according to Sue:

I do give people a fair warning . . . the doctors gonna come in speaking a lot of medical terms . . . I’ll translate for you as best I can . . .

Therefore, a significant issue was the communication gap between autistic mothers and MCPs – the autistic mothers’ ability to express their own needs and understand MCPs, and the MCPs’ ability to express themselves clearly and to understand, accept and relate to the communication from autistic mothers.

### Competing demands and priorities within maternity care

This theme explores the competing priorities that exist for autistic mothers compared to midwives, the system demands experienced by midwives, and how choice is experienced and interpreted within maternity care.

#### Navigating different priorities within maternity care for autistic mothers

The mothers within this study experienced significant negative and long-lasting reactions as a result of medical interactions during antenatal appointments:

She was like measuring and pushing my bump . . . I would never imagine anybody in any circumstance would go pushing like I mean it was sore . . . it really upset me for days afterwards, it’s still upsetting when I think about it. (Alice, mother)

There was a shared understanding of these physical interactions as traumatic and upsetting. Here, Ellen explains how she interpreted the experience of a midwife checking how dilated she was in labour:

It was just monstrous, and she was really brutal and I was trying to kick her off and swearing at her and I had to be pinned down . . . (Ellen, mother)

Interestingly, only the midwives who were on the autism spectrum considered the sensory experience of medical procedures within interviews:

For example, with vaginal examinations . . . it’s terrifying to me, but we do it to women all the time. (Katie, midwife)

Autistic mothers expressed a strong desire for emotional care within the maternity setting. They viewed themselves as vulnerable and felt there was no space for emotional expression within maternity care. ‘Nobody really asked me about anything very much about how I was feeling . . . It was really quite unsettling . . .’ (Ellen, mother, on her pregnancy experience).

This lack of attention to emotions and the sensory experience of medical interactions from the perspective of midwives can be attributed to midwives’ priority of ensuring the physical health of mother and child. It can also be explained by the competing demands midwives experience within the maternity context. Sue demonstrated a keen awareness of autistic mother’s need for additional support but felt constrained by the demands within the system to provide such support:

It could be deemed as . . . you’re spending too much time but . . . the ultimate goal is to make sure you’ve got people who can look after themselves and their baby. (Sue, midwife)

This highlights the difficulty in meeting the individual needs of autistic mothers within an imperfect maternity care system.

#### Interpretations and experiences of choice

The different priorities that exist in maternity care had an impact on how autistic mothers viewed their position as autistic patients within maternity care. Mothers experienced limited choice in decision-making: ‘And I didn’t feel like they saw me as a human being, I was just a subject and one that wasn’t performing as she was supposed to’ (Ellen, mother, on labour in hospital).

Autistic mothers felt their opinions about their own births were not valued and there was a strong sense of having to get in line with professionals’ opinions. This approach of ‘MCP knows best’ was also perceived as a barrier to providing adapted care by the midwives: ‘I know a lot of colleagues are very much like OK, I’m going to tell you what to do and you’re going to do it’ (Sue, midwife).

This perception of limited choice was corroborated by midwives, who believed that any decisions by mothers, that could be considered out of the ordinary, were grounds for judgement from professionals. Ann described, ‘Some women might not consent to like some things that are very routine for everybody else . . . you know it would become a moral judgment . . . like oh, why is she doing that?’

Notably, midwives also experienced some limitations in terms of an opportunity to provide adaptations to maternity care for autistic women. Describing how her attempts to adapt the environment are not valued by other MCPs, Sue said: ‘You’ve got doctors that will still come in and they will whack up the lights to full brightness . . . and like no, come on, it’s not nice’.

Both midwives and mothers shared a perception that midwifery-led care is more amenable to adapting to individual differences. Rebecca experienced a consultant-led model for the birth of her first baby and a midwifery-led model for her second birth experience:

In the hospital, it’s like you’re a number in the queue, aren’t you? It’s get in, have your baby, get out. With the midwife it’s so much more calmer, and it is about you and how you want to do it. (Rebecca, mother)

For both mothers and midwives, there is a sense that midwifery-led care is more person-centred and offers greater choice, which empowers mothers to take control of their own birth experience, whereas limited choice and interpretation of limited ability to share their own maternity experience contribute to a sense of feeling out of control. The perception of limited autonomy by midwives may have meaning within the wider maternity and health care context.

## Discussion

Autistic mothers in this study outlined the difficulty they experienced in navigating maternity care services. This was particularly apparent when the lived experience of maternity care did not meet mothers’ expectations. This is consistent with research on neurotypical populations, which suggests that disparity between experience and expectations is a significant factor in patient satisfaction with care during the perinatal period (Britton, 2012). This discrepancy may be particularly impactful for women on the autism spectrum due to preferences for predictability and certainty, which can be characteristic of the condition (South & Rodgers, 2017; Stark et al., 2021).

The difficulties that autistic mothers experience within maternity care are compounded by the lack of awareness of autism from MCPs, as observed by midwives in the current study. This perceived lack of autism awareness is in keeping with findings from other groups of health care professionals (Morris et al., 2019). Within the current study, this lack of awareness from MCPs meant that autistic mothers experienced an additional challenge of having to identify and communicate their specific needs. This lack of understanding can further contribute to the misperceptions and stigma regarding autism within maternity care, which have been identified here and which support claims made in previous research (Pohl et al., 2020). Notably, although aware of the necessity of medical intervention, autistic mothers in the current study experienced medical interactions requiring physical touch as particularly distressing (Beck, 2009).

### Relational care within the maternity context for autistic mothers

The caring relationship was experienced as a salient feature of maternity care for both autistic mothers and midwives. A consistent research finding for all populations is that the quality of relational support has a significant impact on mothers’ perceptions of perinatal care and has implications for postnatal functioning and the experience of postnatal depression (Britton, 2012; Michels et al., 2013; Tinkler & Quinney, 1998). The establishment of a supportive relationship may be more challenging for cross-neurotype dyads, contributing to a ‘double-empathy’ problem. In other words, this suggests that autistic and non-autistic people may have difficulty in mindreading towards each other compared to others in their neurotype groups (Milton et al., 2016). Recent work on the ‘triple-empathy’ problem (Shaw et al., 2023) proposes further barriers to communication within health care specifically due to cultures and agendas within medical practice. For example, the mothers we interviewed felt the health care professionals had different priorities to them and were less concerned about their well-being. Research by Lum et al. (2014) supports the view that autistic women experience greater challenges within health care communication compared to non-autistic women. However, our findings also highlight that when this supportive relationship is available, it has a profound positive effect on mothers and is experienced as rewarding by midwives. Interestingly, it appears from the reports of the autistic midwives that supportive relationships were quite easily established between autistic mothers and midwives, and the midwives became allies and advocates for their patients. This included explaining the doctor’s communications in clearer terms and protecting the sensory environment by keeping lighting low. It would be interesting to know in further studies whether autistic midwives reveal their own autism status to autistic patients, and how this would be perceived by autistic mothers. Autistic mothers and midwives within the current study believed continuity of care played an important role in building this relationship and identifying or communicating specific challenges faced by autistic mothers. Notably, midwifery continuity of care models have been shown to be associated with a range of improved clinical outcomes for mothers and babies (Bradford et al., 2022), as well as greater satisfaction with maternity care for mothers (Perriman et al., 2018). Our findings further support this and offer dialogical perspectives on this relationship.

### Competing demands and priorities within maternity care

Consistent with previous research undertaken with neurotypical mothers (Britton, 2012), autistic mothers within the current study experienced limited choice, control and participation in decision-making, which impacted how they made sense of their experience of maternity care. This perception of limited choice appears to be a common phenomenon for women in maternity care (Bohren et al., 2015). However, current findings highlight that autistic women may be more likely to attribute this to a problem within themselves rather than a problem within the system, perhaps due to long-held experiences of feeling different (Milner et al., 2019).

Similarly, midwives feel constrained in their ability to adapt maternity care for autistic mothers by the competing demands that exist within the system. Midwives in the current study felt overworked and had limited autonomy in their approach to care. The majority of midwives in the current study worked within the National Health Service (NHS). However, research in an Irish context by Doherty and O’Brien (2022) highlights that midwives experience comparatively high levels of burnout, which has implications for care provision. Organisational factors such as the absence of care pathways, heavy workload, and an absence of continuity of care have been identified as barriers to midwives addressing mental health concerns in the perinatal period (Higgins et al., 2018). Such factors offer some explanation as to why midwives cannot consistently prioritise the needs identified by autistic mothers within the current study.

Both the midwives and autistic mothers in the current study identified that midwifery-led maternity care appeared more suitable for meeting the needs of autistic women. Available research highlights that, for low-risk women, midwifery-led care improves a number of health outcomes, reduces the amount of intervention in labour, and increases patient satisfaction with care (Sutcliffe et al., 2012). Future research could explore how specific aspects of midwifery-led care could be applied across maternity care settings or the feasibility of incorporating midwifery-led care across contexts.

### Clinical implications

These findings show that autistic mothers can experience considerable sensory and interpersonal challenges throughout the perinatal period. In the period leading up to and including birth, sensory challenges can arise from the environment itself (lights, noise) and interpersonal contact (examinations, appointments and labour). Given the individual impact these factors may play in a mother’s ability to navigate the experience, flexible and person-centred approaches to maternity care are welcomed. These could include clearly communicating what procedures are happening and what to expect, considering lighting and noise minimisation and offering this where possible, and facilitating continuity of care to allow trust to develop between the mother and MCP. Educating MCPs about communication differences in autism may also improve the acceptance of autistic mothers and reduce any possible stigma or judgement. Despite the challenges presented by the physical environment, the presence of a supportive, caring relationship appears to be a protective and valued experience. This is in line with findings from a neurotypical population (Britton, 2012) and suggests that midwives, and other MCPs, need to explicitly consider the relationship and work towards strengthening and developing this relationship with autistic mothers.

The lack of awareness of autism within maternity care is particularly apparent in relation to the traumatic meaning of autistic women attributed to physical interventions and the lack of consideration for this aspect of care by midwives. The experience of trauma in the birth environment is associated with long-term trauma symptoms, relationship difficulties, fear of childbirth, and difficulties in the mother-infant relationship (Beck, 2009). Therefore, there is a need for increased awareness and training on autism within maternity care. Increasingly, there is an understanding of the importance of research and training that is led or co-created by autistic people (Pellicano et al., 2014) and evidence to support the importance of peer support for autistic adults in different contexts (Crane et al., 2021). The experiences, views, and unique positions of the autistic midwives within the current study represent an opportunity to build on their practices of advocating for autistic mothers within maternity care.

Notably, autistic mothers’ preferences in relation to the environment, increased availability of choice, continuity of care, and midwifery-led care are shared by many recipients of maternity care (Bohren et al., 2015; Britton, 2012). Such care is not yet widely available but has been linked with improved maternal and infant outcomes (Bradford et al., 2022; O’Brien et al., 2021; Sutcliffe et al., 2012). Therefore, ongoing efforts to improve the experience of maternity care for all are warranted and are likely to hold additional benefits for mothers on the autism spectrum.

### Limitations and future directions

It appears that this study was the first to explore MCP’s experiences of delivering maternity care to autistic mothers. Although recruitment was open to MCPs from any professional background, the study sample was made up solely of midwives, and so may not represent the experiences of other MCPs. Two of the midwives had autism diagnoses, and two had close relatives with autism diagnoses. Therefore, this sample of midwives has a unique ‘insider’ perspective on the experience of autism, which influences their professional experience of delivering maternity care to autistic women. This work further builds on recent literature examining the experience of autistic health care professionals, who can face unique challenges in the health care profession (Doherty et al., 2021; Shaw et al., 2023).

The current research does not fully address the subjective, interpretative experiences of neurotypical midwives delivering maternity to autistic women. Therefore, future research should consider the views of neurotypical midwives. It would also be helpful to consider the views and experiences of MCPs from different backgrounds such as obstetricians, gynaecologists and public health nurses.

The interviews with autistic mothers and midwives were predominantly retrospective recollections of their experiences, some of which were decades ago. Therefore, these accounts may not fully represent current health care practices. Furthermore, their interpretations of these experiences may have changed over time. This is particularly relevant for the sample of autistic mothers, which varied in relation to when they first received an autism diagnosis. Only one of the mothers was aware of her autism diagnosis at the time of pregnancy and labour, while another mother was diagnosed in the postnatal period. Therefore, participants were recounting their experiences with a new understanding of themselves as autistic, which may impact their subjective interpretations over time. Future research with women who know they are autistic at the time of pregnancy and birth would enable further understanding of the experience of disclosing an autism diagnosis to MCPs, the efficacy of autism-centred care planning, and the availability of supports or adaptations for autistic mothers. Most mothers recounted their experience of their first birth, and the accounts may not reflect the experiences of parents with multiple children.

It is also important to note that all of the autistic mothers sampled within the current study did not have intellectual disability and were able to communicate verbally. Therefore, their experiences are not representative of the full spectrum of autism.

Finally, seven of the eight participants were based in the United Kingdom and had experienced a UK model of maternity care. All participants were white European women, and therefore, the sample lacked diversity regarding ethnicity. Future research should consider wider socioeconomic and cultural factors which will also impact on access to support and health care.

## Conclusion

This study has drawn together the perspectives of autistic mothers and midwives to deepen the understanding of the experience of receiving and providing maternity care for mothers on the autism spectrum. Findings highlight the unique challenges autistic mothers experience in navigating the maternity care system and the preferences they hold for maternity care, which aligns in many ways with overall preferences for maternity care. Autism appears to be an overlooked experience in the delivery of maternity care, which leads to misperceptions, difficulty forming the essential caring relationship, and creates the potential for harm to mothers on the autism spectrum. The unique perspectives of autistic midwives within the current study represent an opportunity for changes to the maternity care experience for autistic mothers.

## References

[bibr1-13623613241274518] Beck C. T. (2009). Birth trauma and its sequelae. Journal of Trauma & Dissociation, 10(2), 189–203. 10.1080/15299730802624528 19333848

[bibr2-13623613241274518] Bohren M. A. Vogel J. P. Hunter E. C. Lutsiv O. Makh S. K. Souza J. P. Aguiar C. Saraiva Coneglian F. Diniz A. L. A. Tunçalp Ö. Javadi D. Oladapo O. T. Khosla R. Hindin M. J. Gülmezoglu A. M. (2015). The mistreatment of women during childbirth in health facilities globally: A mixed-methods systematic review. PLOS Medicine, 12(6), Article e1001847. 10.1371/journal.pmed.1001847 PMC4488322 26126110

[bibr3-13623613241274518] Borg Xuereb C. Shaw R. L. Lane D . (2015). Patients’ and physicians’ experiences of atrial fibrillation consultations and anticoagulation decision-making: A multi-perspective IPA design. Psychology & Health, 31(4), 436–455. 10.1080/08870446.2015.1116534 26540308

[bibr4-13623613241274518] Bradford B. F. Wilson A. N. Portela A. McConville F. Fernandez Turienzo C. Homer C. S. E. (2022). Midwifery continuity of care: A scoping review of where, how, by whom and for whom? PLOS Global Public Health, 2(10), Article e0000935. 10.1371/journal.pgph.0000935 PMC10021789 36962588

[bibr5-13623613241274518] Britton J. R. (2012). The assessment of satisfaction with care in the perinatal period. Journal of Psychosomatic Obstetrics & Gynecology, 33(2), 37–44. 10.3109/0167482X.2012.658464 22554136

[bibr6-13623613241274518] Corden K. Brewer R. Cage E. (2021). A systematic review of healthcare professionals’ knowledge, self-efficacy and attitudes towards working with autistic people. Review Journal of Autism and Developmental Disorders, 1(14), 386–399. 10.1007/s40489-021-00263-w

[bibr7-13623613241274518] Crane L. Hearst C. Ashworth M. Davies J. Hill E. L. (2021). Supporting newly identified or diagnosed autistic adults: An initial evaluation of an autistic-led programme. Journal of Autism and Developmental Disorders, 51(3), 892–905. 10.1007/s10803-020-04486-4 32266684 PMC7954714

[bibr8-13623613241274518] Department of Health. (2010). Fulfilling and rewarding lives: The strategy for adults with autism in England. https://webarchive.nationalarchives.gov.uk/20130104203954/http://www.dh.gov.uk/en/Publicationsandstatistics/Publications/PublicationsPolicyAndGuidance/DH_113369

[bibr9-13623613241274518] Dern S. Sappok T. (2016). Barriers to healthcare for people on the autism spectrum. Advances in Autism, 2(1), 2–11. 10.1108/AIA-10-2015-0020

[bibr10-13623613241274518] Doherty J. O’Brien D. (2022). Reducing midwife burnout at organisational level – Midwives need time, space and a positive work-place culture. Women and Birth, 35(6), e563–e572. 10.1016/j.wombi.2022.02.003 35181238

[bibr11-13623613241274518] Doherty M. Johnson M. Buckley C. (2021). Supporting autistic doctors in primary care: Challenging the myths and misconceptions. The British Journal of General Practice, 71(708), 294–295. 34319879 10.3399/bjgp21X716165 PMC8249017

[bibr12-13623613241274518] Donovan J. (2020). Childbirth experiences of women with autism spectrum disorder in an acute care setting. Nursing for Women’s Health, 24(3), 165–174. 10.1016/j.nwh.2020.04.001 32389581

[bibr13-13623613241274518] Donovan J. Chiatti B. D. McKeever A. Bloch J. R. Gonzales M. S. Birati Y. (2023). ‘Yes, I can bond’”. Reflections of autistic women’s mothering experiences in the early postpartum period. Women’s Health, 19, 17455057231175312. 10.1177/17455057231175312 PMC10201178 37209090

[bibr14-13623613241274518] Gardner M. Suplee P. D. Bloch J. Lecks K. (2016). Exploratory study of childbearing experiences of women with Asperger syndrome. Nursing for Women’s Health, 20(1), 28–37. 10.1016/j.nwh.2015.12.001 26902438

[bibr15-13623613241274518] Hampton S. Allison C. Aydin E. Baron-Cohen S. Holt R. (2022). Autistic mothers’ perinatal well-being and parenting styles. Autism, 26(7), 1805–1820. 10.1177/13623613211065544 35105233 PMC9483197

[bibr16-13623613241274518] Hampton S. Allison C. Baron-Cohen S. Holt R . (2024). Autistic people’s perinatal experiences i: a survey of pregnancy experiences. Journal of Autism and Developmental Disorders, 54(1), 211–223. 36261629 10.1007/s10803-022-05754-1 PMC10791798

[bibr17-13623613241274518] Higgins A. Downes C. Monahan M. Gill A. Lamb S. A. Carroll M. (2018). Barriers to midwives and nurses addressing mental health issues with women during the perinatal period: The Mind Mothers study. Journal of Clinical Nursing, 27(9–10), 1872–1883. 10.1111/jocn.14252 29314366

[bibr18-13623613241274518] INVOLVE. (2012). Briefing notes for researchers: Involving the public in NHS, public health and social care research. https://www.nihr.ac.uk/documents/briefing-notes-for-researchers-public-involvement-in-nhs-health-and-social-care-research/27371

[bibr19-13623613241274518] Larkin M. Shaw R. Flowers P. (2019). Multi-perspectival designs and processes in interpretative phenomenological analysis research. Qualitative Research in Psychology, 16(2), 182–198. 10.1080/14780887.2018.1540655

[bibr20-13623613241274518] Litchman M. L. Tran M. J. Dearden S. E. Guo J. W. Simonsen S. E. Clark L. (2019). What women with disabilities write in personal blogs about pregnancy and early motherhood: Qualitative analysis of blogs. JMIR Pediatrics and Parenting, 2(1), Article e12355. 10.2196/12355 PMC6715048 31518332

[bibr21-13623613241274518] Loomes R. Hull L. Mandy W. P. L. (2017). What is the male-to-female ratio in autism spectrum disorder? A systematic review and meta-analysis. Journal of the American Academy of Child & Adolescent Psychiatry, 56(6), 466–474. 10.1016/j.jaac.2017.03.013 28545751

[bibr22-13623613241274518] Lum M. Garnett M. O’Connor E. (2014). Health communication: A pilot study comparing perceptions of women with and without high functioning autism spectrum disorder. Research in Autism Spectrum Disorders, 8(12), 1713–1721. 10.1016/j.rasd.2014.09.009

[bibr23-13623613241274518] MacLeod A. (2019). Interpretative Phenomenological Analysis (IPA) as a tool for participatory research within Critical Autism Studies: A systematic review. Research in Autism Spectrum Disorders, 64, 49–62. 10.1016/j.rasd.2019.04.005

[bibr24-13623613241274518] McDonnell C. G. DeLucia E. A. (2021). Pregnancy and parenthood among autistic adults: Implications for advancing maternal health and parental well-being. Autism in Adulthood, 3(1), 100–115. 10.1089/aut.2020.0046 36601267 PMC8992883

[bibr25-13623613241274518] Michels A. Kruske S. Thompson R. (2013). Women’s postnatal psychological functioning: The role of satisfaction with intrapartum care and the birth experience. Journal of Reproductive and Infant Psychology, 31(2), 172–182. 10.1080/02646838.2013.791921

[bibr26-13623613241274518] Milner V. McIntosh H. Colvert E. Happé F. (2019). A qualitative exploration of the female experience of autism spectrum disorder (ASD). Journal of Autism and Developmental Disorders, 49(6), 2389–2402. 10.1007/s10803-019-03906-4 30790191 PMC6546643

[bibr27-13623613241274518] Milton D. Martin N. Melham P. (2016). Beyond reasonable adjustment: Autistic-friendly spaces and Universal Design. Pavilion Publishing and Media.

[bibr28-13623613241274518] Misra D. P. Guyer B. Allston A. (2003). Integrated perinatal health framework. American Journal of Preventive Medicine, 25(1), 65–75. 10.1016/S0749-3797(03)00090-4 12818312

[bibr29-13623613241274518] Morris R. Greenblatt A. Saini M. (2019). Healthcare providers’ experiences with autism: A scoping review. Journal of Autism and Developmental Disorders, 49(6), 2374–2388. 10.1007/s10803-019-03912-6 30758692

[bibr30-13623613241274518] National Disability Authority. (2014). Annual report. http://nda.ie/Publications/Others/National-Disability-Authority-Annual-Reports/Annual-Report-2014.html

[bibr31-13623613241274518] O’Brien D. Butler M. M. Casey M. (2021). The importance of nurturing trusting relationships to embed shared decision-making during pregnancy and childbirth. Midwifery, 98, 102987. 10.1016/j.midw.2021.102987 33761433

[bibr32-13623613241274518] Pellicano E. Dinsmore A. Charman T. (2014). What should autism research focus upon? Community views and priorities from the United Kingdom. Autism, 18(7), 756–770. 10.1177/1362361314529627 24789871 PMC4230972

[bibr33-13623613241274518] Perriman N. Davis D. L. Ferguson S. (2018). What women value in the midwifery continuity of care model: A systematic review with meta-synthesis. Midwifery, 62, 220–229. 10.1016/j.midw.2018.04.011 29723790

[bibr34-13623613241274518] Pohl A. L. Crockford S. K. Blakemore M. Allison C. Baron-Cohen S. (2020). A comparative study of autistic and non-autistic women’s experience of motherhood. Molecular Autism, 11(1), 3. 10.1186/s13229-019-0304-2 31911826 PMC6945630

[bibr35-13623613241274518] Rogers C. Lepherd L. Ganguly R. Jacob-Rogers S. (2017). Perinatal issues for women with high functioning autism spectrum disorder. Women and Birth, 30(2), e89–e95. 10.1016/j.wombi.2016.09.009 27751685

[bibr36-13623613241274518] Rostill-Brookes H. Larkin M. Toms A. Churchman C. (2011). A shared experience of fragmentation: Making sense of foster placement breakdown. Clinical Child Psychology and Psychiatry, 16(1), 103–127. 10.1177/1359104509352894 20538717

[bibr37-13623613241274518] Samuel P. Yew R. Y. Hooley M. Hickey M. Stokes M. A. (2022). Sensory challenges experienced by autistic women during pregnancy and childbirth: A systematic review. Archives of Gynecology and Obstetrics, 305(2), 299–311. 10.1007/s00404-021-06109-4 34085111

[bibr38-13623613241274518] Shaw S. C. Fossi A. Carravallah L. A. Rabenstein K. Ross W. Doherty M. (2023). The experiences of autistic doctors: A cross-sectional study. Frontiers in Psychiatry, 14, Article 1160994. 10.3389/fpsyt.2023.1160994 PMC10393275 37533891

[bibr39-13623613241274518] Smith J. A. Flowers P. Larkin M. (2009). Interpretative phenomenological analysis: Theory, method and research. Sage.

[bibr40-13623613241274518] Smith J. A. Nizza I. E. (2022). Essentials of interpretative phenomenological analysis. American Psychological Association.

[bibr41-13623613241274518] South M. Rodgers J. (2017). Sensory, emotional and cognitive contributions to anxiety in autism spectrum disorders. Frontiers in Human Neuroscience, 11, Article 20. 10.3389/fnhum.2017.00020 PMC5258728 28174531

[bibr42-13623613241274518] Stark E. Stacey J. Mandy W. Kringelbach M. L. Happé F. (2021). Autistic cognition: Charting routes to anxiety. Trends in Cognitive Sciences, 25(7), 571–581. 10.1016/j.tics.2021.03.014 33958281

[bibr43-13623613241274518] Sundelin H. E. Stephansson O. Hultman C. M. Ludvigsson J. F. (2018). Pregnancy outcomes in women with autism: A nationwide population-based cohort study. Clinical Epidemiology, 10, 1817–1826. 10.2147/CLEP.S176910 30555264 PMC6280895

[bibr44-13623613241274518] Sutcliffe K. Caird J. Kavanagh J. Rees R. Oliver K. Dickson K. Woodman J. Barnett-Paige E. Thomas J. (2012). Comparing midwife-led and doctor-led maternity care: A systematic review of reviews. Journal of Advanced Nursing, 68(11), 2376–2386. 10.1111/j.1365-2648.2012.05998.x 22489571

[bibr45-13623613241274518] Talcer M. C. Duffy O. Pedlow K. (2023). A qualitative exploration into the sensory experiences of autistic mothers. Journal of Autism and Developmental Disorders, 53, 834–849. https://doi.org/10.1007/s10803-021-05188-1? 34251566 10.1007/s10803-021-05188-1 PMC9944021

[bibr46-13623613241274518] Tinkler A. Quinney D. (1998). Team midwifery: The influence of the midwife-woman relationship on women’s experiences and perceptions of maternity care. Journal of Advanced Nursing, 28(1), 30–35. 10.1046/j.1365-2648.1998.00769.x 9687127

[bibr47-13623613241274518] Unigwe S. Buckley C. Crane L. Kenny L. Remington A. Pellicano E. (2017). GPs’ confidence in caring for their patients on the autism spectrum: An online self-report study. British Journal of General Practice, 67(659), e445–e452. 10.3399/bjgp17X690449 PMC5442960 28483821

[bibr48-13623613241274518] Urbanowicz A. Parkin T. Dooren K. van Girdler S. Ciccarelli M. Lennox N. (2020). The experiences, views, and needs of health professionals who provide care to adults on the autism spectrum. Research and Practice in Intellectual and Developmental Disabilities, 7, 179–192. 10.1080/23297018.2020.1735943

[bibr49-13623613241274518] Zerbo O. Massolo M. L. Qian Y. Croen L. A. (2015). A study of physician knowledge and experience with autism in adults in a large integrated healthcare system. Journal of Autism and Developmental Disorders, 45(12), 4002–4014. 10.1007/s10803-015-257 26334872

